# Ultrasound innovations in abdominal radiology: multiparametic imaging in liver transplantation

**DOI:** 10.1007/s00261-024-04518-y

**Published:** 2024-08-21

**Authors:** Samantha S. Chau, Bryce D. Beutler, Edward G. Grant, Hisham Tchelepi

**Affiliations:** 1 Department of Radiology, University of Southern California, Keck School of Medicine, Los Angeles, CA USA; 2 Department of Radiology, Los Angeles General Medical Center, Los Angeles, CA USA

**Keywords:** Abdominal ultrasound, Contrast-enhanced ultrasound, Liver transplant, Ultrasound

## Abstract

**Purpose:**

Ultrasound plays a central role in liver transplant evaluation. Acute, subacute, and chronic complications can be readily identified using grayscale and color Doppler ultrasound. Contrast-enhanced ultrasound adds a new dimension to liver transplant evaluation, depicting vascular and parenchymal processes with exquisite detail. In addition, emerging evidence suggests that contrast-enhanced ultrasound may allow for localization of biliary leak in select patients. We aimed to assess the use of multiparametric ultrasound—including grayscale, color and spectral Doppler, and contrast-enhanced ultrasound—in the setting of liver transplantation.

**Methods:**

A literature review was performed using the MEDLINE bibliographic database through the National Library of Medicine. The following terms were searched and relevant citations assessed: “abdominal ultrasound,” “contrast-enhanced ultrasound,” “liver transplant,” and “ultrasound.”

**Results:**

Grayscale and color Doppler ultrasound represent the mainstay imaging modalities for postoperative liver transplant evaluation. The addition of contrast enhancement plays a complementary role and can provide valuable information related to the allograft vasculature, parenchyma, and biliary tree. The appropriate implementation of grayscale, color Doppler, and contrast-enhanced ultrasound can optimize sensitivity, specificity, and accuracy for the detection of liver transplantation complications, including hepatic artery stenosis, biliary leakage, and infection.

**Conclusion:**

Multimodal sonographic evaluation is essential to identify postoperative complications in liver transplant recipients. Contrast-enhanced ultrasound may be of value in challenging cases, providing excellent anatomic delineation and reducing the risk of false-positive and false-negative diagnoses. A broad familiarity with appropriate applications of both nonenhanced and contrast-enhanced ultrasound may help radiologists optimize allograft assessment and improve patient outcomes.

## Introduction

Ultrasound plays a central role in liver transplant evaluation, representing the mainstay imaging modality for assessment of the graft parenchyma, biliary tree, and vasculature. Acute complications of liver transplantation, including perihepatic hematoma and arterial thrombosis, are readily identified using conventional grayscale and color Doppler ultrasound. Delayed complications—including biliary stricture, hepatic arterial stenosis, and allograft rejection—are also frequently diagnosed on postoperative ultrasound. Contrast-enhanced ultrasound (CEUS) has further improved sonographic evaluation of liver transplants, providing exquisitely sensitive flow visualization in the hepatic artery and portal vein and allowing for early detection of vascular complications [1]. In addition, emerging evidence suggests CEUS may improve localization of biliary leakage and characterization of biliary strictures in select patients.

In this review, we describe the use of multiparametric ultrasound for liver transplant evaluation with a special emphasis on clinical applications of CEUS. In addition, we provide case examples from our institution that illustrate the advantages of ultrasound for the diagnosis of both common and atypical liver transplant complications. The preponderance of the evidence together with our clinical experience suggests that multiparametric ultrasound is invaluable for the detection of both early and late postoperative liver transplantation complications.

## Discussion

### Vascular

Liver transplantation involves the creation of multiple vascular anastomoses. The surgical approach is informed by both donor and recipient related factors, including the size of the donor allograft and the native hepatic arterial anatomy. An end-to-end hepatic arterial anastomosis between the donor common hepatic artery and recipient proper hepatic artery, described as a “fish mouth” anastomosis, is the most commonly employed technique [2, 3]. However, the presence of variant hepatic arterial anatomy or donor-recipient size mismatch may necessitate more complex reconstructions; an aortohepatic conduit or double anastomosis is often used for recipients with replaced or accessory hepatic arteries [4, 5]. Caval, portal, and hepatic venous anastomotic techniques may also vary by donor and recipient anatomy. A piggyback technique involving in situ anastomosis of the donor and recipient inferior vena cava with a concomitant end-to-end portal venous anastomosis represents the most common surgical approach, but a bicaval technique or interposition graft from the superior mesenteric vein may be required in some individuals [6].

Vascular complications may occur in relation to an anastomosis or proximal or distal to the anastomotic site. Hepatic artery stenosis is the most common postoperative arterial complication, occurring in 4 to 11% of transplant recipients, and nearly always occurs at or near an anastomosis [7]. The underlying cause is likely multifactorial and related to intraoperative trauma, vascular kinking, and extrinsic compression [8]. Inflammatory processes in the setting of acute cellular rejection may also play a contributory role in some patients. Hepatic artery stenosis may occur any time after transplantation, but most commonly develops within the first year, with a median time to diagnosis of three months [9].

The hallmark sonographic finding of hepatic artery stenosis is a diminished resistive index below 0.5 and a tardus parvus waveform, characterized by prolonged systolic acceleration and diminished systolic amplitude with rounding of the systolic peak [10]. Dodd et al*.* suggested that three quantitative parameters may be used to establish a probable diagnosis of arterial stenosis based on Doppler ultrasound waveform: (1) resistive index less than 0.5; (2) systolic acceleration time longer than 0.08 s; and (3) peak systolic velocity greater than 200 cm/sec at the anastomosis [11]. In a subsequent study by Park et al*.*, authors aimed to establish an optimal peak systolic velocity threshold for the diagnosis of hepatic artery stenosis. The Park group found that, in the presence of a tardus parvus waveform, a peak systolic velocity less than or equal to 48 cm/sec was 69% sensitive and 99% specific for hepatic artery stenosis [12]. Other authors have proposed that more stringent criteria should be used to increase specificity. In a 2018 analysis by Zheng et al., authors showed that using a resistive index less than 0.4 and a systolic acceleration time longer than 0.12 s significantly decreased the false-positive rate without increasing the false-negative rate in patients with hepatic arterial stenosis [13]. However, a low resistive index and tardus parvus waveform can also be seen in the setting of hepatic arterial thrombosis and arterioportal fistula, among other etiologies, and direct sonographic visualization of the stenosis at or near the anastomosis is essential to establish a definitive diagnosis.

Direct visualization of a hepatic artery stenosis can be challenging. A peak systolic velocity greater than 200 cm/sec at the anastomosis can be used to identify the area of stenosis, but the specific site and degree of stenosis can be difficult to ascertain based on Doppler ultrasound alone given that the stenosis often lies outside the liver and may be challenging to visualize. The application of CEUS may be of value to delineate the type, degree, and specific sites of stenosis, which may inform endovascular intervention [14]. In addition, limited data suggest that CEUS is more sensitive than grayscale and color Doppler ultrasound for the detection of early low-grade arterial stenosis [15]. At our institution, CEUS is commonly used to confirm suspected hepatic artery stenosis and depict the type, length, and number of stenoses for preprocedural planning.

Hepatic arterial thrombosis represents the second most common arterial complication in the early postoperative setting, with an incidence of approximately 4.4% reported in the medical literature [16]. Notably, however, the incidence of perioperative hepatic arterial thrombosis at our institution has progressively decreased over time and is now relatively rare. Delayed hepatic arterial thrombosis occurs months to years after transplantation and is somewhat less common, but may occur in up to 1.7% of recipients [17]. Irrespective of the temporal onset, the mortality rate and risk of allograft loss is high; up to one-third of patients who develop hepatic arterial thrombosis will die and over half will require a repeat transplant. However, early diagnosis significantly increases the likelihood of successful revascularization and allograft salvage [18].

The sensitivity and specificity of grayscale and color Doppler ultrasound for hepatic arterial thrombosis varies based on the time frame relative to surgery. In the immediate and early postoperative period, the reported sensitivity and specificity of ultrasound may be as high as 92% and 88%, respectively [19–21]. However, sensitivity is significantly lower in the setting of delayed or late hepatic arterial thrombosis, likely due to collateralization that confounds the sonographic assessment of vascular flow [22].

The classic sonographic findings of early hepatic arterial thrombosis follow a relatively predictable pattern, which was first described by Nolten and Sproat and has since been confirmed in several analyses [19]. The earliest sign of early hepatic arterial thrombosis is diminished diastolic flow, manifesting as an elevated resistive index. A resistive index of 0.8 is commonly used as a cutoff value, although correlation with laboratory values and sonographic follow-up is required to distinguish early thrombosis from expected postoperative edema. There is subsequently dampening of the systolic peak, which will ultimately progress to complete loss of hepatic arterial flow. Late hepatic arterial thrombosis presents with distinct sonographic findings due to the common presence of arterial collaterals. Sonographic features are similar to hepatic artery stenosis; a resistive index below 0.5 and tardus parvus waveforms are classically observed in these cases. In both early and late hepatic arterial thrombosis, the complete absence of vascular flow is typically considered diagnostic (see Table 1).

**Table 1:** Clinical applications of conventional and contrast-enhanced ultrasound for liver transplant evaluation

Complication	Conventional ultrasound	Contrast-enhanced ultrasound
Hepatic arterial stenosis	Dodd criteria Tardus parvus waveform RI < 0.5 Systolic acceleration time > 0.08 s PSV > 200 cm/s at the anastomosis Park threshold PSV ≤ 48 cm/s in the presence of a tardus parvus waveform	Identification of the specific type, length, and number of hepatic arterial stenoses
Hepatic arterial thrombosis	Early postoperative period Diminished diastolic flow with an RI > 0.8 Dampening of the systolic peak Complete loss of hepatic arterial flow Late postoperative period Tardus parvus waveform RI < 0.5 Complete loss of hepatic arterial flow	Early enhancement of the portal vein Non-enhancement of the intrahepatic and extrahepatic arterial supply Abrupt cutoff of the affected hepatic artery Allograft infarcts
Hepatic artery pseudoaneurysm	Saccular outpouching arising from the hepatic artery with bidirectional flow in a “yin-yang” pattern To-and-fro spectral waveform	Delineation of the vasculature to distinguish a true pseudoaneurysm from the expected fish-mouth arterial anastomosis Preprocedural identification of the pseudoaneurysm neck
Portal vein stenosis	Preanastomotic-to-anastomotic velocity ratio of 1:3 Velocity change > 60 cm/sec across an anastomosis Velocity greater than 125 cm/sec at an anastomosis	Localize and evaluate the degree of stenosis Distinguish high-grade portal vein stenosis from thrombosis Assess for allograft hypoperfusion
Portal vein thrombosis	Echogenic thrombus within the portal vein and its tributaries Internal vascularity in the setting of tumor in vein	Identify tumor in vein, characterized by arterial phase enhancement and delayed phase washout within the thrombus Detect small thrombi in peripheral portal venous branches
Hepatic vein torsion	Flow reversal within the hepatic vein	Evaluation for focal or wedge-shaped non-enhancement, indicating parenchymal hypoperfusion
Biliary leakage	Avascular, predominantly anechoic intrahepatic or perihepatic collection	Localization of biliary leaks
Biliary stricture	Intrahepatic and extrahepatic biliary ductal dilatation with onre or multiple areas of abrupt narrowing Small, irregular biliary tree outpouchings in the setting of nonanastomotic strictures	Assessment for active biliary epithelial and periductal inflammation, characterized by arterial phase hyperenhancement and delayed phase hypoenhancement
Hepatic abscess	Complex, predominantly hypoechoic collection, often containing gas and echogenic debris	Distinguish hepatic infarct, biloma, and abscess; only a hepatic abscess shows arterial phase rim enhancement or a honeycomb apperance with avid enhancement of intralesional septae
Hepatic neoplasm	Hepatocellular carcinoma – hypoechoic mass with internal vascular flow and a peripheral hyperechoic halo PTLD – well-defined echogenic mass with a peripheral hypoechoic rim and low vascular flow	LI-RADS can be used to establish a diagnosis of hepatocellular carcinoma Pattern of enhancement may be used to distinguish opportunistic infection from PTLD

*LI-RADS* liver imaging reporting and data system, *PTLD* post-transplant lymphoproliferative disease, *RI* resistive index

Caution must be applied in regard to elevated resistive indices in the early postoperative period, as an elevated resistive index alone is a relatively common and self-limited finding of no clinical consequence. In the late postoperative period, an elevated resistive index may indicate an intrinsic parenchymal process, such as rejection, and should be interpreted with consideration of the clinical context. Progressive dampening of the systolic peak with eventual loss of arterial flow is a more specific finding that is highly-suggestive of thrombosis.

Grayscale and color Doppler ultrasound represent the first-line imaging modalities to assess for hepatic arterial thrombosis in both symptomatic and asymptomatic patients. However, although the sensitivity and specificity are high, false-positive and false-negative findings are not uncommon, particularly in the late postoperative setting. Common causes of false-positive cases include hepatic parenchymal edema, vasospasm, and systemic hypotension, which are frequently present in the early postoperative period [23]. High-grade hepatic artery stenosis can also mimic thrombosis. False-negative findings most often result from the development of periportal collateral arteries, which are inadequate to perfuse the liver parenchyma and intrahepatic biliary tree but produce a normal Doppler waveform that masks underlying thrombosis [22, 24].

A delayed diagnosis of hepatic arterial thrombosis can have devastating clinical consequences. The emergence of CEUS represents a paradigm shift in the evaluation of suspected hepatic arterial thrombosis, allowing for diagnosis with strikingly high sensitivity, specificity, and accuracy. Indeed, in a 2012 prospective study by Lu et al*.*, authors found that CEUS provided a sensitivity, specificity, and accuracy of 100, 96.9, and 92.9% for diagnosing hepatic arterial thrombosis [25]. A subsequent study by Kim et al*.* established that CEUS shows higher specificity and positive predictive value than computed tomography angiography (CTA) for the diagnosis of hepatic arterial thrombosis [26].

**Fig. 1:**
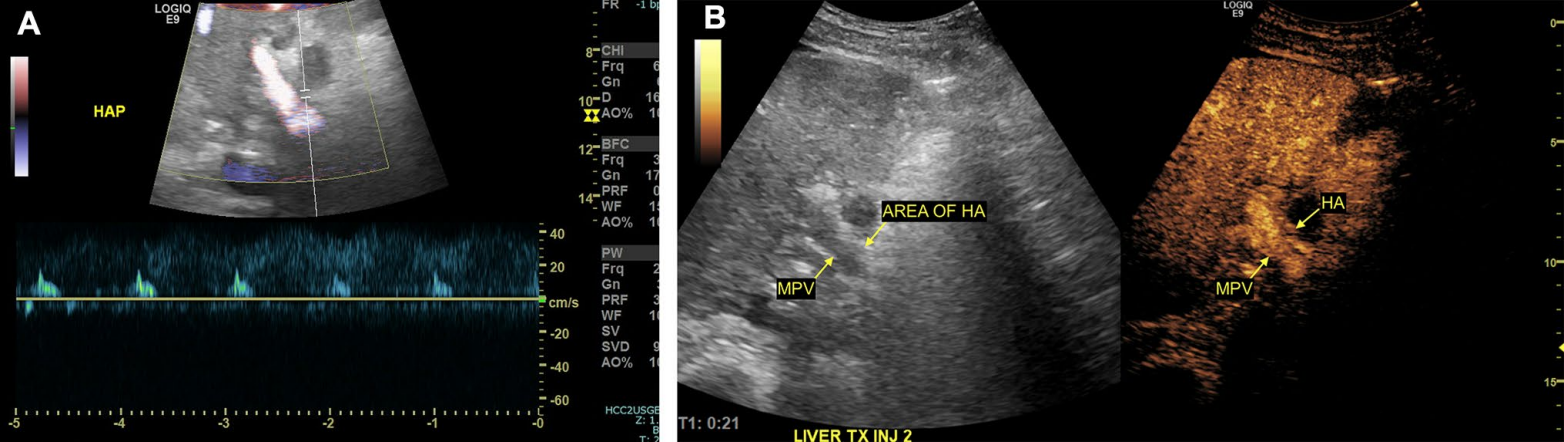
Spectral Doppler (**a**) and contrast-enhanced (**b**) ultrasound demonstrating hepatic artery thrombosis in a 65-year-old male who presented with clinical and laboratory findings concerning for diminished arterial flow one day after liver transplantation. Spectral Doppler showed a “water-hammer” arterial waveform, characterized by normal peak systolic velocity with a sharp decline and flow reversal during diastole. Contrast-enhanced ultrasound demonstrated early portal venous enhancement with delayed enhancement of a diminutive hepatic artery with an abrupt cutoff proximal to the expected bifurcation (arrow). Hepatic arterial thrombosis was subsequently confirmed intra-operatively

**Fig. 2:**
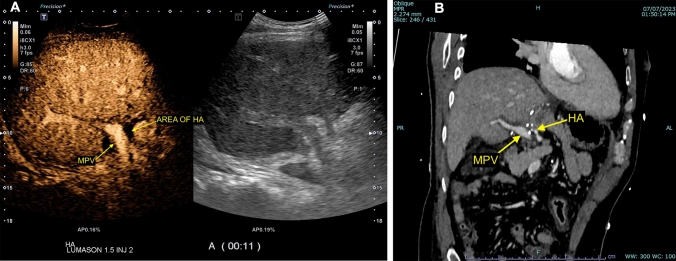
Contrast-enhanced ultrasound (**a**) and contrast-enhanced computed tomography (CT) scan (**b**) showing hepatic artery thrombosis in a 33-year-old male who presented for sonographic assessment of the allograft five days after liver transplantation. The contrast-enhanced ultrasound showed avid early enhancement of the portal venous system with absent intrahepatic arterial enhancement, compatible with proximal thrombosis. Proper hepatic artery thrombosis was subsequently confirmed on CT scan

A presumptive diagnosis of hepatic arterial thrombosis is typically confirmed using CEUS at our institution. Classic findings with CEUS are easily interpreted and include absent enhancement of the intrahepatic and extrahepatic arterial supply with early enhancement of the portal vein; in the setting of a patent hepatic artery, the arteries should enhance before the portal vein, whereas in thrombosis, the portal vein will be the only vessel showing enhancement (Figs. 1 and 2). In some cases, an abrupt cutoff of the affected hepatic artery can be seen, allowing for localization of the thrombus. Hepatic parenchymal infarcts are frequently present and will show absent enhancement on arterial, portal venous, and delayed phases. The presence of hepatic arterial enhancement virtually excludes the possibility of thrombosis, preventing unnecessary angiography or surgery. Advantages of CEUS over CTA and magnetic resonance angiography (MRA) include the ability to perform dynamic imaging, allowing for multiple acquisitions and direct interrogation of areas of concern over the course of a scan, which aids in problem-solving in challenging cases. In addition, there is no ionizing radiation exposure associated with sonography, and microbubble contrast agents have few contraindications and can be safely administered to patients with renal failure or iodinated contrast allergies.

Hepatic artery pseudoaneurysms are rare, occurring in only 0.3 to 3% of transplant recipients, but may be catastrophic [27]. The mortality rate has been reported to be as high as 2 to 3% and up to 70% in the setting of rupture [28]. The majority of pseudoaneurysms develop within the extrahepatic arterial supply and are caused by underlying infection, which may occur secondary to colonization of the subhepatic space by enteric pathogens in the setting of Roux-en-Y hepaticojejunostomy. Other recognized risk factors for extrahepatic pseudoaneurysms include untreated hepatic artery stenosis, bile leak, fungal infection, adhesions, and technical factors related to surgery [29, 30]. Intrahepatic pseudoaneurysms are relatively rare and typically represent iatrogenic injuries related to prior procedures, such as liver biopsy or percutaneous biliary drainage [31]. Complications of untreated pseudoaneurysms include rupture and fistulization, either of which may manifest with rapid decompensation and death [32]. Assessment for pseudoaneurysm therefore represents a priority in the postoperative setting.

**Fig. 3:**
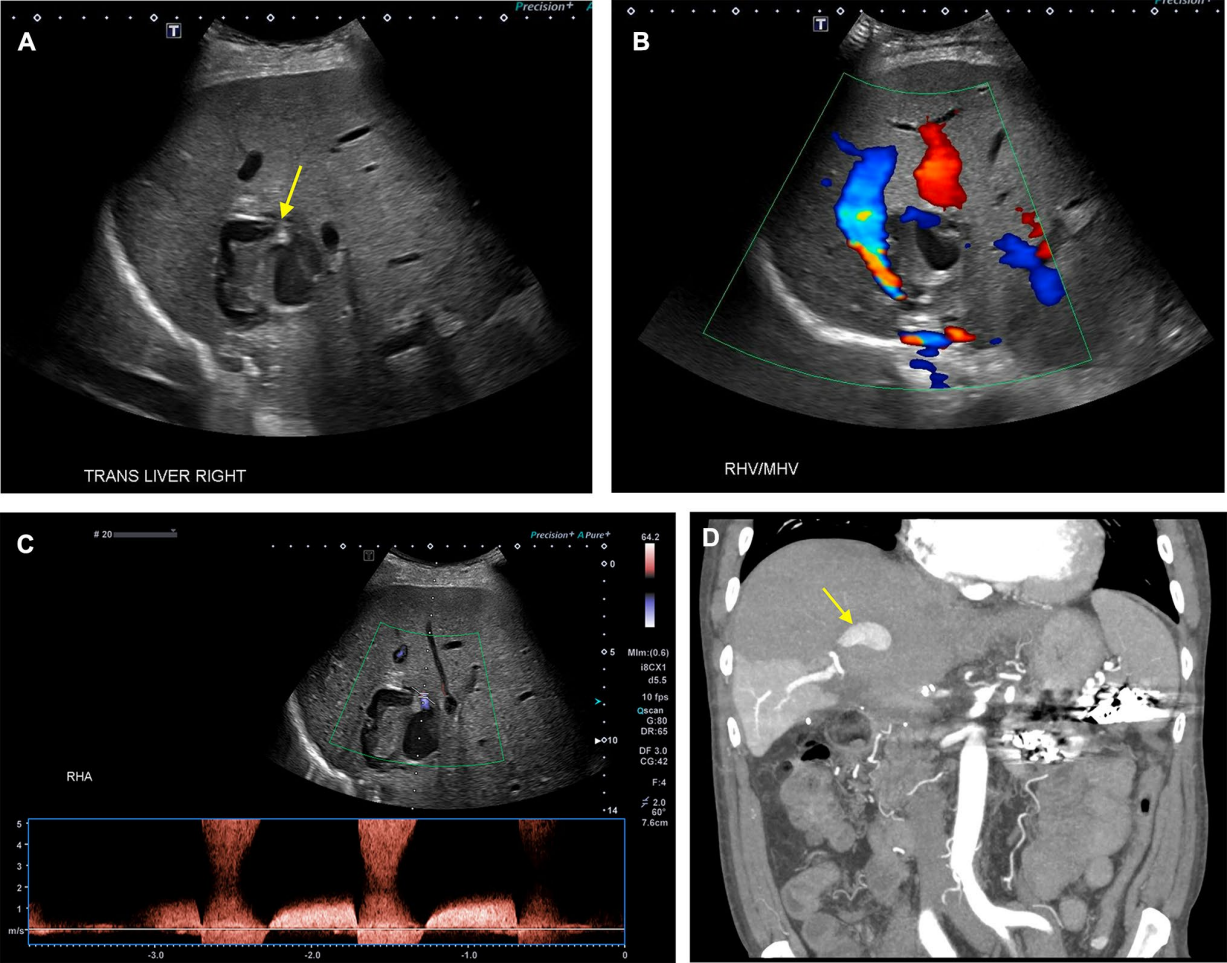
Grayscale (**a**), color Doppler (**b**), spectral Doppler (**c**), and computed tomography angiography (**d**) demonstrating a partially thrombosed hepatic artery pseudoaneurysm in a 66-year-old male with a history of orthotopic liver transplantation who presented with anemia three weeks after a percutaneous liver biopsy. Grayscale and color Doppler showed a small, irregular saccular outpouching with vascular flow arising from the hepatic artery (**a** and **b**). Spectral Doppler showed an avascular collection with a “to-and-fro” arterial waveform, characterized by markedly elevated arterial velocity with reversal of flow in diastole (**c**). The suspected pseudoaneurysm was subsequently confirmed on computed tomography angiography (**d**)

Ultrasound is the first-line imaging modality to assess for hepatic artery pseudoaneurysm. Grayscale ultrasound typically reveals a rounded or saccular outpouching arising from the hepatic artery. Color Doppler ultrasound classically shows local bidirectional flow, often referred to as the “yin-yang sign,” which is caused by turbulence within the pseudoaneurysmal sac. A corresponding “to-and-fro” spectral waveform pattern indicating blood entering the pseudoaneurysmal sac during systole and exiting the sac during diastole is typically present (Fig. 3).

Conventional ultrasound is highly-specific for the diagnosis of hepatic artery pseudoaneurysm. However, sensitivity is limited. In one study by Kim et al*.*, authors reported that a hepatic artery pseudoaneurysm was detected in only one of eight patients using grayscale and color Doppler ultrasound [30]. Other authors have described similarly low sensitivity for extrahepatic pseudoaneurysms, although the detection rate of intrahepatic pseudoaneurysms is reportedly significantly higher, likely due to the improved sonic window [33]. False-positives are relatively uncommon, but may occur if the focally dilated appearance of a fish mouth anastomosis is mistaken for an aneurysmal sac.

The sensitivity of ultrasound for hepatic artery pseudoaneurysms is considerably higher with contrast enhancement. In one retrospective study by Ren et al*.*, investigators reported that sensitivity increased from 37.5% with conventional ultrasound to 75% with CEUS, rivaling that of CTA [34]. Contrast enhancement is also of value for delineating the contour of the fish mouth anastomosis, reducing the likelihood of false-positive findings. In addition, hepatic artery pseudoaneurysms may occasionally develop in association with the anastomosis; CEUS provides superior anatomic detail to identify the pseudoaneurysm origin for treatment planning. At our institution, CEUS is performed for patients with a clinical presentation concerning for pseudoaneurysm but normal or equivocal sonographic findings.

**Fig. 4:**
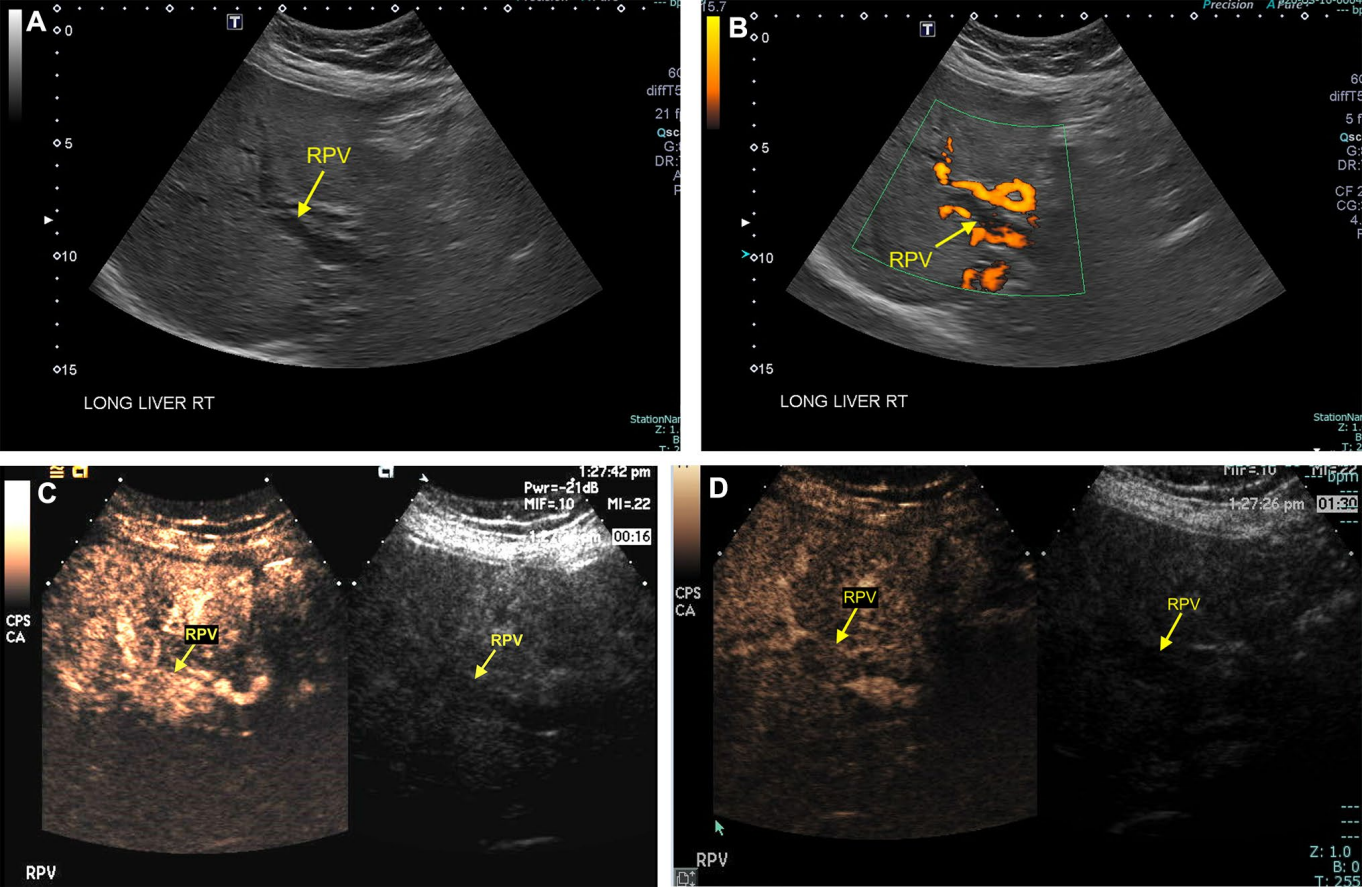
Grayscale (**a**), power Doppler (**b**), and contrast-enhanced (**c** and **d**) ultrasound showing portal vein thrombosis in a 69-year-old male with a history of hepatocellular carcinoma who presented with abdominal pain six months after liver transplantation. Laboratory studies showed transaminitis and elevated serum alpha-fetoprotein. Grayscale ultrasound demonstrated a heterogeneous echotexture of the liver parenchyma (**a**). Power Doppler ultrasound demonstrated normal flow within the main and left portal veins with nearly occlusive echogenic thrombus within the right portal vein (**b**). A focused ultrasound showed avid early enhancement within the right portal vein thrombus that was significantly greater than that of the surrounding liver parenchyma (**c**) with delayed washout (**d**), consistent with tumor in vein

Portal venous and caval complications are relatively uncommon. However, venous thrombosis and stenosis may occur in the early and late postoperative setting. The incidence of portal vein thrombosis in liver transplant recipients ranges from 0.3 to 2.6%, with thrombosis most commonly occurring within three months of surgery [35, 36]. The clinical symptomatology is variable; thrombosis in the early postoperative period typically manifests with fulminant allograft dysfunction whereas delayed thrombosis presents with sequelae of portal hypertension, such as portosystemic collateralization and ascites. Grayscale and color Doppler ultrasound is 89% sensitive and 92% specific for a diagnosis of portal vein thrombosis [37]. However, although conventional sonography is highly accurate, emerging evidence suggests that CEUS may serve as an important adjunct in equivocal cases. In a retrospective analysis by Rennert et al*.*, authors reported that CEUS was more sensitive than grayscale and color Doppler ultrasound alone, correctly diagnosing portal vein thrombosis in cases that were previously interpreted as negative [38]. CEUS can also be used to identify small thrombi in peripheral portal venous branches and quantify the severity of portal venous insufficiency by depicting the degree and pattern of parenchymal hypoperfusion [39]. In addition, hepatocellular carcinoma may rarely develop within an allograft, and CEUS can help distinguish bland thrombus from tumor in vein, the latter of which shows avid arterial enhancement with delayed phase washout (Fig. 4) [40].

Portal vein stenosis is rare and almost always occurs at the site of an end-to-end anastomosis or at the anastomosis of an interposition jump graft from the superior mesenteric vein. Sonographic findings may include a preanastomotic-to-anastomotic velocity ratio of 1:3 or a velocity change of greater than 60 cm/sec across an anastomosis. A velocity of greater than 125 cm/sec at an anastomosis is also highly suggestive of portal vein stenosis [41]. However, elevated velocity at an anastomosis is very common in the early postoperative period secondary to edema and other non-pathologic processes, and serial sonographic follow-up may be indicated to distinguish normal perioperative findings from true stenosis. Progression from portal vein stenosis to complete thrombosis may occur if left untreated. Grayscale and color Doppler ultrasound are typically adequate to establish a diagnosis of portal vein stenosis, but CEUS may be of value to distinguish stenosis from thrombosis and identify the specific location of stenosis, the degree of pre-stenotic dilatation, and the presence hepatic parenchymal hypoperfusion [38]. Management may involve venoplasty with balloon dilatation or stenting.

Caval and hepatic vein complications may occur in up to 3% of transplant recipients. A piggyback anastomosis between the donor and recipient inferior vena cava is a common site of stenosis, which can be identified on ultrasound by the presence of a monophasic waveform, venous pulsatility index lower than 0.45, and a three-fold or greater increase in velocity across the anastomosis [42]. A modified piggyback technique using a cuff of three suprahepatic veins has reportedly been associated with a lower risk of stenosis, although stenosis may still occur and will manifest with sonographic findings similar to those seen in a conventional piggyback technique. Hepatic vein and inferior vena cava thrombosis typically occur as a result of intraoperative trauma, vessel kinking, or in relation to an underlying hypercoagulable state. Sonographic findings include a monophasic waveform or reversal of flow with CEUS showing absent or decreased contrast enhancement [15]. Hepatic vein torsion is an exceedingly rare cause of venous outflow obstruction resulting from a significant donor-recipient allograft size mismatch or inadequate hepatopexy that is characterized by flow reversal or hepatic parenchymal hypoperfusion on CEUS [43].

### Biliary

Biliary complications occur in up to 30% of patients undergoing liver transplantation [44]. Bile leakage is the most common complication in the early postoperative period, with an incidence of up to 25%, and typically occurs at the ductal anastomosis [45, 46]. The risk of bile leakage may vary based on the type of biliary reconstruction performed during surgery; limited evidence suggests that a Roux-en-Y choledochojejunostomy is associated with a significantly greater risk of postoperative biliary leakage as compared to an end-to-end ductal anastomosis or duct-to-duct choledochocholedochostomy [47]. Bile leakage may also occur from a cystic duct stump or at the ductal insertion of a T-tube in patients who require temporary external biliary drainage [48].

Ultrasound is the first-line imaging modality to assess for biliary leakage. Grayscale and color Doppler ultrasound typically reveal an avascular, predominantly anechoic intrahepatic or perihepatic collection. The term “biloma” is commonly used when the collection is organized or loculated. Assessment of echogenicity can be valuable to distinguish a biloma from other early postoperative complications; although bilomas frequently contain echogenic debris, a completely anechoic collection likely represents a biloma whereas a complex or mixed echogenicity collection is suggestive of hematoma or abscess. Nevertheless, it can be challenging to distinguish a gas-containing biloma from a hepatic abscess. Moreover, bilomas can become superinfected. Other tools that may be of value to distinguish a biloma from an abscess include CEUS; the presence of arterial phase rim enhancement or a honeycomb appearance with enhancement of intralesional septae suggests a diagnosis of abscess [49]. In addition, the presence of hepatic artery stenosis favors a diagnosis of abscess over biloma. However, careful correlation of the unenhanced ultrasound findings, CEUS, and the clinical context is required to establish a presumptive diagnosis. Nuclear medicine cholescintigraphy using an iminodiacetic acid analog or magnetic resonance imaging with a hepatocyte-specific contrast agent, such as gadoxetate disodium, is required for confirmation.

There is emerging evidence suggesting that CEUS may allow for localization of biliary leakage in select patients. In a case report by Mao et al*.*, investigators used CEUS to identify the site of biliary leak in a patient with recent T-tube removal [50]. The Mao group performed ultrasound-guided percutaneous drainage of a presumed biloma and subsequently injected a microbubble contrast agent through the catheter. The CEUS delineated the biliary tree and localized the biliary leak to the common bile duct, helping to facilitate endoscopic treatment. Other authors have used CEUS to localize biliary leakage at an anastomosis. In a 2018 review by Huang et al*.*, authors described a sonographic technique in which administration of a microbubble ultrasound contrast agent through a drainage catheter allowed for precise localization of a biliary leak with pooling of the contrast agent near the anastomosis [51].

Biliary strictures represent the most common biliary complication of liver transplantation and may occur months to years after surgery [52]. Strictures are broadly classified into two distinct categories. Anastomotic strictures are relatively common, involve the extrahepatic bile duct, and are easily treated with endoscopic balloon dilation and stent placement with a low risk of allograft loss [53]. Nonanastomotic strictures, in contrast, typically develop secondary to an underlying pathologic process, such as infection, ischemia, or recurrence of underlying primary sclerosing cholangitis, tend to be multifocal, are difficult to treat, and have been associated with a high risk of allograft loss. Nonanastomotic strictures also tend to present earlier in the postoperative course relative to anastomotic strictures and are usually diagnosed within six months of transplantation, manifesting with progressive transaminitis and hyperbilirubinemia [54].

Intrahepatic and extrahepatic biliary ductal dilatation with abrupt narrowing is the sonographic hallmark of a biliary stricture. Multifocal or long segment narrowing is often present in the setting of nonanastomotic strictures. Small, irregular biliary tree outpouchings can occasionally be seen in the setting of nonanastomotic strictures secondary to recurrence of primary sclerosing cholangitis. The use of CEUS may be of value to assess for active biliary epithelial and periductal inflammation, which is characterized by arterial phase hyperenhancement and delayed phase hypoenhancement [55]. Ultrasound is typically adequate to establish a diagnosis of anastomotic or nonanastomotic strictures, but magnetic resonance cholangiopancreatography (MRCP) is often useful for confirmation and further anatomic delineation.

Biliary ductal necrosis and biliary cast syndrome are uncommon liver transplantation complications that occur secondary to hypoperfusion. The bile ducts are supplied solely by the hepatic artery and thus severe stenosis or thrombosis may have catastrophic downstream effects on the biliary tree [56]. The sonographic features of biliary ductal necrosis are nonspecific and may include only diffuse intrahepatic biliary ductal dilatation; the presence of biliary ductal dilatation in the absence of an obvious stricture should therefore prompt interrogation of the hepatic artery for thrombosis or stenosis. Perfusion of the biliary tree can be further assessed with CEUS, which may show biliary wall hypoenhancement in the setting of ischemia [57].

Biliary cast syndrome is readily identified on grayscale ultrasound. Early biliary cast syndrome is characterized by small periportal branching structures that are isoechoic to the surrounding liver parenchyma, which represent bile ducts that are slightly distended by sludge. Progression of biliary cast syndrome will present with hyperechoic filling defects comprised of necrotic biliary mucosa, which distend the intrahepatic and extrahepatic bile ducts. Untreated biliary cast syndrome may result in the development of biliary leakage or stricture due to progressive inflammation and necrosis [58]. There is no established role for CEUS in the setting of biliary cast syndrome.

### Parenchymal

Parenchymal complications of liver transplantation may occur any time over the lifespan of an allograft. Infectious and ischemic complications tend to occur in the early postoperative period and can be reliably diagnosed using grayscale and color Doppler ultrasound with application of CEUS for challenging cases. A hepatic abscess appears as a poorly-circumscribed, complex, predominantly hypoechoic collection, often containing internal gas and hyperechoic debris. Acute hepatic infarction may appear as an ill-defined, avascular hypoechoic region with indistinct borders. Internal locules of gas with associated dirty shadowing can often be seen in the setting of necrosis. The presence of hepatic artery thrombosis supports the diagnosis. The infarcted tissue will become progressively more anechoic or cystic and well-defined over time.

**Fig. 5:**
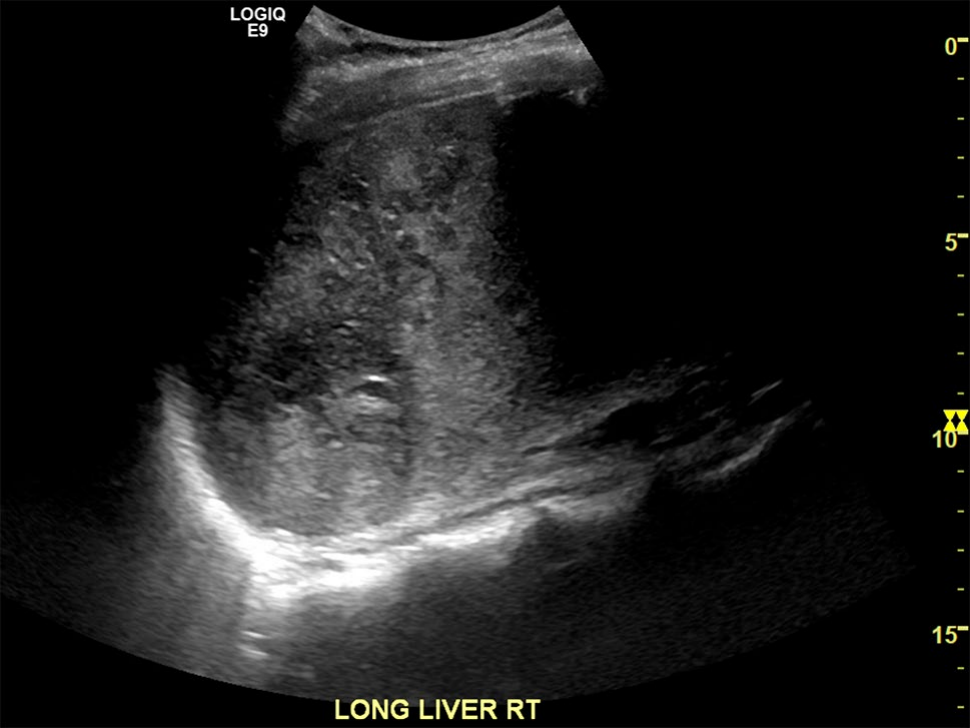
Grayscale ultrasound illustrating the “bright band” sign, characterized by heterogeneously hypoechoic liver parenchyma with interposed linear echogenic bands, which are thought to represent specular reflections from the intact portal triads. The bright band sign is relatively specific for hepatic infarction and can be used to distinguish infarcted parenchyma from abscess, neoplasm, and other insidious processes

**Fig. 6:**
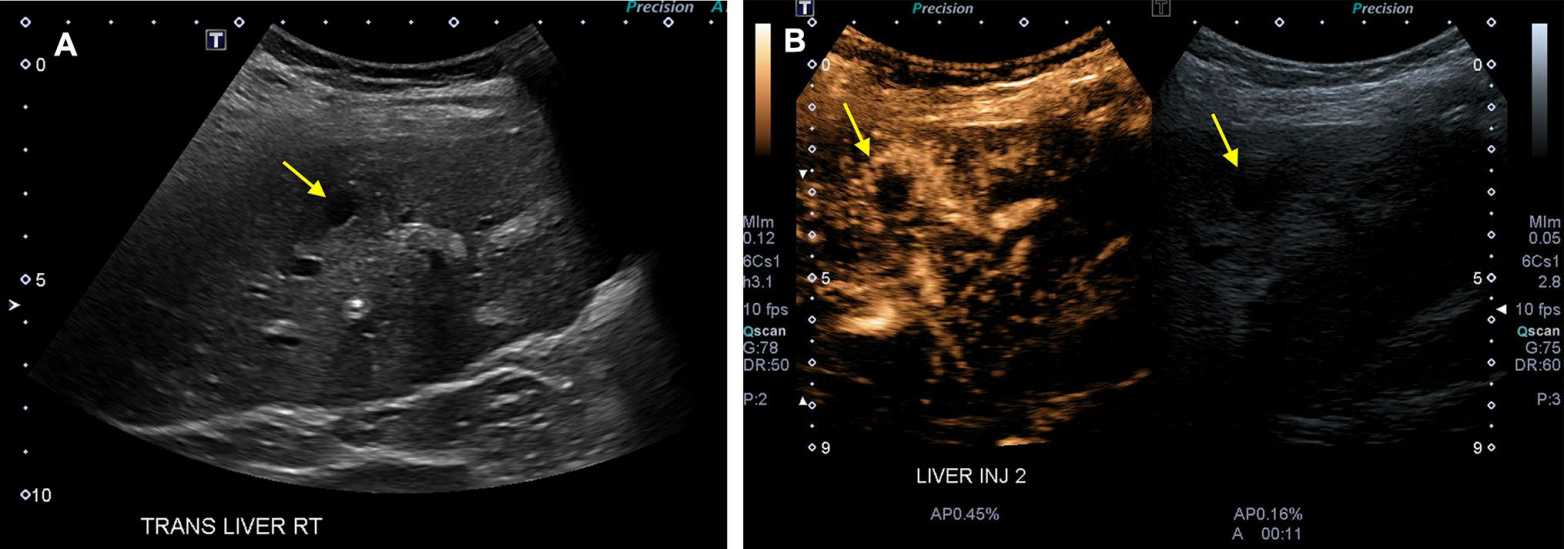
Grayscale (**a**) and contrast-enhanced (**b**) ultrasound showing a hepatic abscess in a 67-year-old male who presented with fever and leukocytosis ten days after liver transplantation. Grayscale and color Doppler ultrasound demonstrated a hypoechoic structure measuring up to 3.2 cm in diameter within the right hepatic lobe with trace internal vascular flow. Contrast-enhanced ultrasound showed avid late arterial phase rim enhancement (acquired at 2 min and 50 s following contrast administration). A diagnosis of cholangitic abscess was suspected and subsequently treated with ultrasound-guided percutaneous drainage

A gas-containing hepatic abscess is often indistinguishable from acute parenchymal infarction on grayscale and color Doppler ultrasound. However, the “bright band” sign, defined as multiple linear echogenic bands traversing a geographic region of hypoechogenicity, is highly suggestive of infarction rather than abscess [59, 60] (Fig. 5). In addition, CEUS can reliably differentiate the two entities. A hepatic abscess demonstrates arterial phase rim enhancement or a honeycomb appearance with avid enhancement of intralesional septae (Fig. 6) [49]. Acute infarction, in contrast, demonstrates hypoenhancement relative to the surrounding liver parenchyma on both arterial and delayed phases [15]. Hepatic abscess and parenchymal infarction require entirely different management strategies and thus correlation of the “bright band” sign and CEUS can significantly improve patient outcomes in the appropriate clinical setting.

**Fig. 7:**
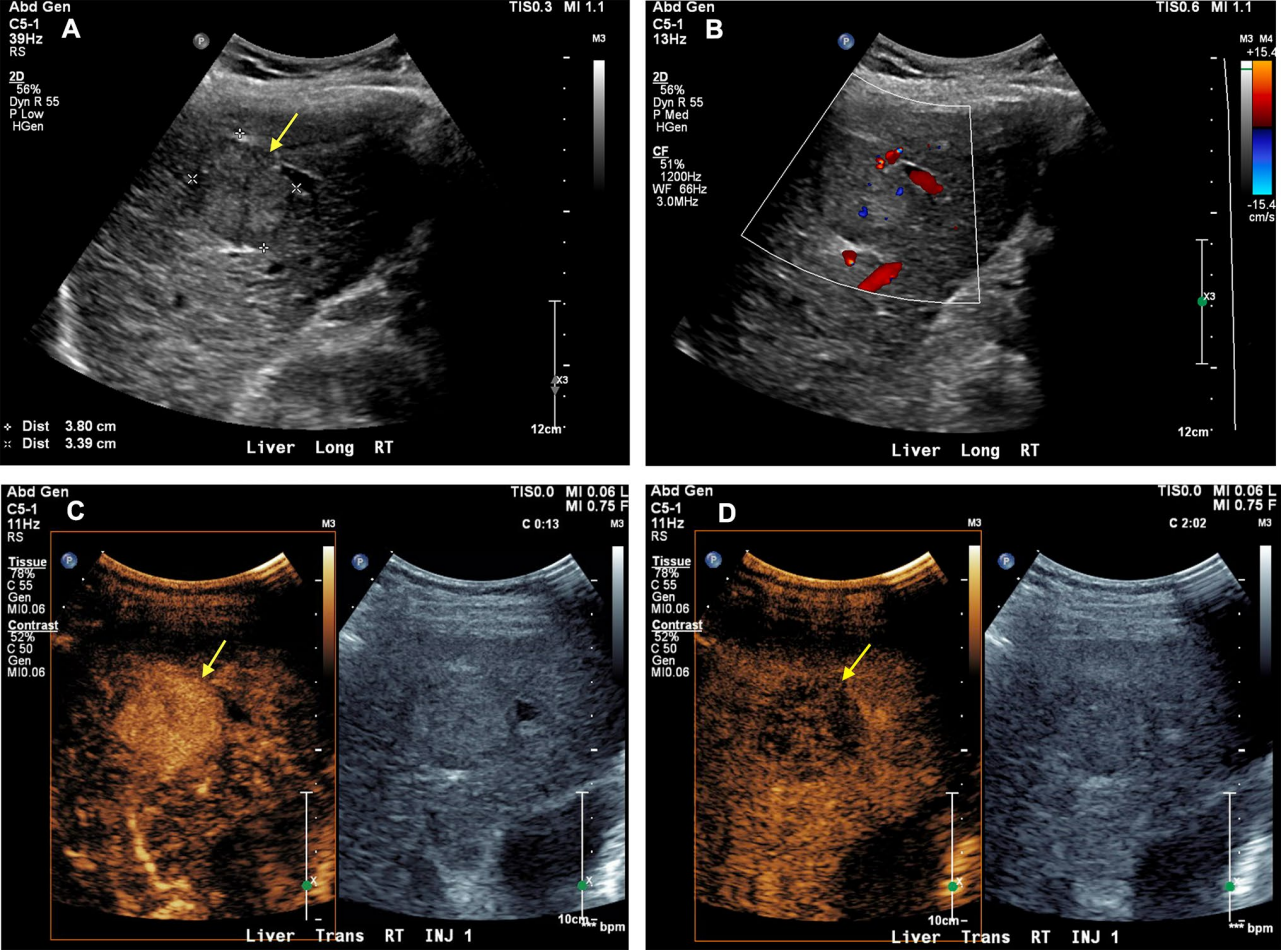
Grayscale (**a**), color Doppler (**b**), and contrast-enhanced (**c** and **d**) ultrasound showing hepatocellular carcinoma in a 61-year-old male who presented for surveillance two years after liver transplantation. Grayscale and color Doppler ultrasound showed a hypoechoic mass measuring 3.6 cm in diameter with internal vascular flow within the right hepatic lobe adjacent to the gallbladder (**a** and **b**). Contrast-enhanced ultrasound showed non-rim arterial phase hyperenhancement (**c**) with late mild washout (**d**), typical of hepatocellular carcinoma (Contrast-Enhanced Ultrasound Liver Imaging Reporting and Data System [CEUS LI-RADS] 5)

Neoplastic complications of liver transplantation occur in the late postoperative period and may include hepatocellular carcinoma, cholangiocarcinoma, and post-transplant lympholiferative disorder (PTLD). Hepatocellular carcinoma classically presents as a hypoechoic mass with internal vascular flow and a peripheral hyperechoic halo. However, many benign and malignant neoplasms, such as focal nodular hyperplasia and hepatic adenoma, may demonstrate similar sonographic features. Recent clinical data suggest that CEUS is highly-specific for the diagnosis of hepatocellular carcinoma [61]. Indeed, the *American College of Radiology* recently endorsed a Liver Imaging Reporting and Data System (LI-RADS) specific to CEUS [62]. At our institution, CEUS has been used to distinguish hepatocellular carcinoma from focal nodular hyperplasia and other parenchymal masses (Fig. 7).

The diagnosis of PTLD can be challenging on account of its variable sonographic appearance. A well-defined echogenic mass with a peripheral hypoechoic rim and low vascular flow is classic, but lesions may also be hypoechoic or isoechoic without detectable vascular flow. One or multiple lesions may be present [63]. Notably, opportunistic infections, such as candidiasis, may mimic PTLD on ultrasound and affect a similar patient population. Emerging data suggest that CEUS may be useful to distinguish PTLD from other infectious and neoplastic processes. In a 2021 case report, Chen et al*.* described the use of CEUS in an immunocompromised patient who presented with multiple unusual, avascular hypoechoic hepatic nodules [64]. The nodules demonstrated a unique enhancement pattern with early homogeneous or heterogeneous arterial enhancement followed by gradual washout during portal venous phase. Other authors have reported applications of CEUS to evaluate for PTLD at other sites [65]. Although CEUS can not be used to establish a definitive diagnosis of PTLD, it appears to be useful to exclude infection and other sonographic mimics.

### Limitations of multiparametic ultrasound

Multiparametric ultrasound is invaluable for the assessment of early and late postoperative complications related to liver transplantation. However, there are several inherent limitations. Clinical findings concerning for perioperative hemorrhage should be urgently assessed with multiphase computed tomography (CT) scan, which is superior to ultrasound for the detection and localization of active bleeding. In addition, although color and spectral Doppler ultrasound represents the first-line modality to screen for hepatic arterial stenosis, CTA is often required for confirmation and preprocedural planning.

The biliary tree can be characterized using unenhanced ultrasound with or without contrast enhancement. However, MRCP is typically necessary to delineate the entirety of the biliary tree, assess for multifocal strictures, and identify secondary causes of obstruction. Cross-sectional imaging may also be required for the assessment of large bilomas that extend beyond the sonographic field-of-view, particularly if there is concern for superinfection.

The data pertaining to multiparametic ultrasound for the assessment of post-transplant neoplastic processes, including PTLD, are somewhat limited. Positron emission tomography-computed tomography (PET-CT) is preferred for the characterization of most neoplasms and to assess for distant metastatic disease. Multiparametic ultrasound is highly sensitive and specific to evaluate for hepatocellular carcinoma using CEUS LI-RADS. However, although ultrasound may be useful for the initial assessment of other malignancies, it but should be considered a screening tool in most cases.

## Conclusion

Ultrasound represents the mainstay imaging modality for the assessment of vascular, biliary, and parenchymal complications following liver transplantation. The addition of CEUS may provide valuable additional information to aid in prognostication and guide management. The continued evolution of advanced sonographic techniques and technologies, including contrast enhancement, will play an important role in transplant evaluation for the foreseeable future.

## Data Availability

No datasets were generated or analysed during the current study.
